# Defining occult injuries of the distal forearm and wrist in children

**DOI:** 10.1007/s11832-016-0735-7

**Published:** 2016-04-27

**Authors:** Michael Elvey, S. Patel, Erez Avisar, W. J. White, E. Sorene

**Affiliations:** Trauma and Orthopaedic Department, University College London Hospital, 250 Euston Road, London, NW1 2PG UK; Trauma and Orthopaedic Department, Royal Free London NHS Foundation Trust, Wellhouse Lane, Barnet, Hertfordshire EN5 3DJ UK; The Hand Surgery Unit, Asaf Haroffeh Medical Centre, POB 70300, Zerifin, Israel

**Keywords:** Paediatric wrist injury, Wrist trauma, Occult wrist injury

## Abstract

**Purpose:**

The nonspecific terms “wrist sprain” and “suspected occult bony injury” are frequently documented as diagnoses in occult paediatric wrist injuries. To date, however, no one has accurately defined their true underlying pathology. The primary objective of this study was to identify the true pathoanatomy of occult acute paediatric wrist injuries. Our secondary objective was to compare our findings with existing adult data in order to determine any population differences that might be clinically relevant.

**Methods:**

We performed a single-centre retrospective case series evaluating MRI findings in acute paediatric wrist injuries presenting to the hand injury unit between 2011 and 2014. All patients underwent standardised radiographs of the wrist and, where clinically indicated, of the scaphoid. Where no bony anomaly was identified, MRI scanning was offered. Cohen’s kappa coefficient was used to calculate the agreement between clinical and MRI diagnosis.

**Results:**

57 patients met the final inclusion criteria. Occult fractures and bony contusions comprised the majority of the pathologies, at 36.5 and 35.0 %, respectively. There were no cases of isolated soft-tissue injury. MRI effected management change in 35.1 % of cases. Paediatric wrists demonstrated differences in injury pattern and distribution when compared to an adult population.

**Conclusion:**

This study defines for the first time the true pathology of occult paediatric wrist injuries. The current definition of a wrist sprain was not applicable to a single case and therefore appears to be inappropriate for use in the paediatric population. A precise knowledge of the likely pathology facilitates accurate information delivery whilst reducing parental uncertainty and treatment variation.

## Introduction

The first radiological sign of wrist trauma in the paediatric patient may be evidence of a healing process. This provides a diagnostic challenge at first presentation for treating clinicians. The terms “wrist sprain” or “occult bony injury” are frequently used, but they are also nonspecific and no one has accurately defined the underlying pathology of these injuries to date. Whilst the majority of such patients recover uneventfully, a small number are at risk of long-term pain and disability secondary to missed diagnoses and inappropriate treatment. The current definition of a wrist sprain is “a partial ligament injury of the wrist without positive plain radiographic findings” [1]. In children, it is accepted that the physis is the weakest part of the musculoskeletal system, and ligamentous injuries are less common than in adults. These observations raise several clinical questions, including the appropriateness of the term “wrist sprain” in a paediatric population, and how the anatomic pathology of occult wrist injuries differs between the paediatric and adult populations.

The primary objective of this study was to identify the true anatomic pathology of acute paediatric wrist injuries presenting with negative initial radiographs. Our secondary objective was to compare our findings with similar existing adult population data in order to determine any differences in the two populations that might be relevant to clinicians dealing with acute trauma.

## Methods

We performed a single-centre retrospective case series evaluating MRI findings in acute paediatric wrist injuries that were presented to the hand injury unit between 2011 and 2014. Institutional review board approval was sought and obtained. Inclusion criteria included age <16 years, presentation to the hand injury unit for wrist pain within 2 weeks of acute trauma, and presentation of negative radiographs. Exclusion criteria included presentation 2 weeks or more following trauma, a clear fracture or dislocation on plain radiographs, and refusal to undergo MRI. All patients were assessed by a consultant hand and wrist surgeon and underwent standardised radiographs of the wrist. Scaphoid radiographs were requested where anatomical snuff box or scaphoid tubercle tenderness was present, and comprised standard wrist posteroanterior (PA) and lateral views, with additional oblique and PA/ulnar deviated views. These images were reported by a consultant musculoskeletal radiologist and re-reviewed by the same hand and wrist consultant. In patients where no bony anomaly could be identified, MRI scanning was offered. MRI scans were performed in a 1.5-T whole-body scanner with a wrist coil. The MRI protocol included coronal T1 spin echo, coronal short tau inversion recovery, coronal T2 gradient echo and axial proton density fat-saturated sequences. Data were recorded on the initial diagnosis after history, examination, and radiographs, final diagnosis after MRI scanning, method of initial treatment, method of final treatment after MRI scanning, and complications. Cohen’s kappa coefficient was used to calculate the agreement between clinical and MRI diagnosis, with the strength of agreement graded according to Landis and Koch’s [2] criteria. Initial management was determined by a combination of suspected clinical diagnosis and symptom severity. Suspected soft-tissue injuries were treated in a removable splint. Suspected occult fractures were treated according to symptom severity. Patients with mild to moderate symptoms were treated in a removable splint. Patients with severe symptoms were treated in a below-elbow plaster of Paris (POP) cast.

## Results

A total of 91 MRI scans were requested in 91 patients in the defined study period. 34 patients presented at >2 weeks following injury, giving a final study population of 57 (29 males and 28 females). The median age of the study population was 12 years (range 8–16), with presentation to the hospital occurring at a median of 5 days (range 0–14) following injury. Thirty-four (60 %) injuries were sustained to the dominant hand. There were no cases of bilateral injury. The median time between injury and MRI was 6 days (range 0–25). The most common mechanism of injury was a fall onto an outstretched hand (*n* = 47, 82.5 %), 39 (83 %) of which were sustained during sporting activity. Seven (12.3 %) patients sustained hyperextension injuries, with two (3.5 %) patients suffering a crush injury and one (1.8 %) patient suffering blunt trauma. On examination, 24 (42.1 %) patients were tender over the distal radius, 11 (19.3 %) in the anatomical snuffbox, seven (12.3 %) had global wrist tenderness, and six (10.5 %) demonstrated ulnar-sided wrist tenderness.

**Fig. 1:**
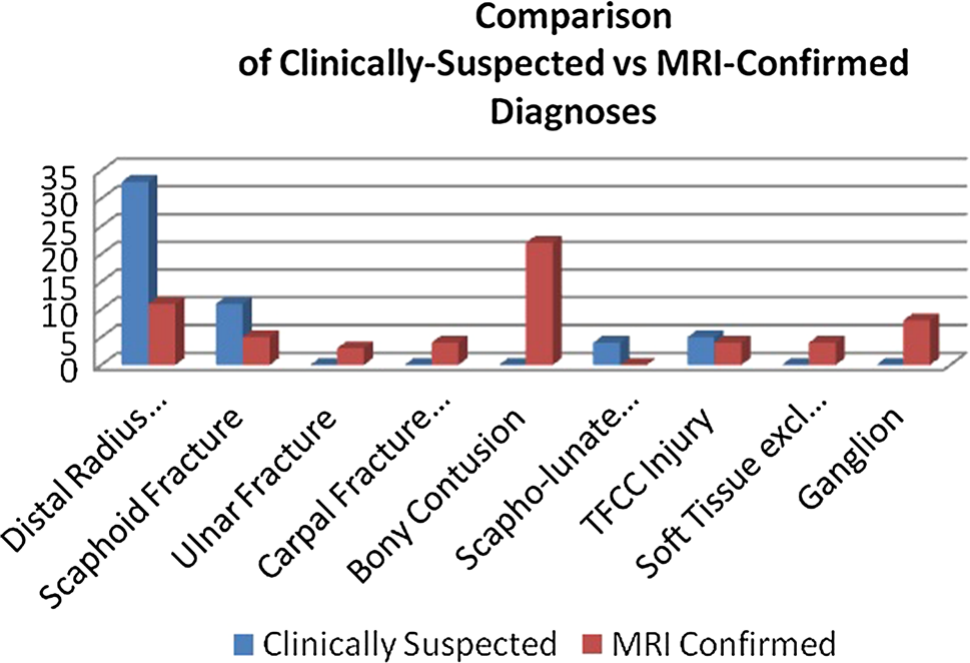
Comparison between clinical and MRI diagnoses

Initial clinical diagnoses included occult distal radius fracture (*n* = 33, 57.9 %), occult scaphoid fracture (*n* = 13, 22.3 %), and soft-tissue injury/wrist sprain (*n* = 17, 29.8 %). Initial management comprised a removable splint (*n* = 35, 61.1 %), POP (*n* = 20, 35.1 %), and analgesia alone (*n* = 2, 3.6 %). No patients declined or were unable to tolerate the MRI scan. 43 patients (75.4 %) had a positive finding on MRI, with a median of 1 positive finding (range 1–3) per patient on imaging. Paediatric MRI findings are detailed in Table 1. A comparison between clinical and MRI diagnoses is depicted in Fig. 1, and a comparison between our paediatric findings and previously published adult data is provided in Table 2.

**Table 1:** Paediatric MRI findings

Finding on MRI	Number
Fracture	
Distal radius	11
Scaphoid	
Distal pole	3
Waist	3
Trapezoid	1
Lunate	1
Ulnar styloid	3
Contusion	
Distal radius	9
Scaphoid	7
Ulnar styloid	2
Trapezoid	3
Lunate	1
Soft-tissue injury	
TFCC injury	
Sprain	3
Tear	1
Tenosynovitis	2
DRUJ injury	1
Extrinsic sprain	1
Ganglion	8

**Table 2:** Comparison between paediatric and adult MRI findings

	Current study	Bergh et al. [13]	Pierre-Jerome et al. [12]
No. patients	57	155	125
Median age (range)	12 (8–16)	28 (18–49)	n/a (adult)
F:M	1:1	1:1.2	1:1.3
Overall incidence OBI (%)	69.6	71.0	62.4
Median positive findings/pt	1 (0–3)	2 (0–8)	(2–6)^a^
Occult fracture			
Total	23 (36.5 %)	44 (28.0 %)	29 (23.2 %)
% Distal radius	52.2	25.9	38
% Scaphoid	26.1	25.9	31
% Ulnar styloid (Fig. 5)	13	3.7	n/a
% Lunate	3.4	0	n/a
% Trapezoid	3.4	1.9	n/a
% Triquetrum	0	11.1	n/a
% Pisiform	0	0	n/a
% Trapezium	0	1.2	n/a
% Capitate	0	8	n/a
% Hamate	0	0	n/a
Bone contusion			
Total	22 (35 %)	33 (22 %)	49 (39 %)
% Distal radius	40.1	12.5	23.7
% Scaphoid	31.2	16.1	26.7
% Ulnar styloid	9.1	3.5	n/a
% Lunate	4.5	10.7	22.1
Soft tissue			
Total	8 (14.5 %)	41 (26.4)	n/a
% TFCC injury	50	34.1	n/a
% Scapholunate injury	0	11.2	n/a
% Partial tendon rupture	0	15.9	n/a
% Tenosynovitis	25	13.6	n/a
% DRUJ injury	12.5	n/a	n/a
% Extrinsic sprain	12.5	18.8	n/a

*OBI* occult bony injury

^a^No median provided

## Discussion

The findings of this study suggest that children presenting with occult wrist injuries have a high incidence of positive findings on MRI scans. Occult bony injuries including fractures and bony contusions are common and differ in pattern and distribution from those in adults. Soft-tissue injuries are always associated with occult bony injuries, so the current definition of wrist sprain appears to be inappropriate for use in the paediatric population.

Fracture lines in children are often absent on initial radiographs, and the earliest radiological signs of trauma may relate to healing in the form of callus or sclerosis 4–5 weeks following injury. This has raised the question of the appropriate choice and timing of imaging modality. Bone scintigraphy and ultrasound are both available to assist in the detection of occult fractures; however, despite excellent sensitivity, the former lacks specificity and the latter is highly operator-dependent [3]. Significant differences in patient management have been shown to result following cross-sectional imaging, and the relative merits of computed tomography (CT) scanning versus MRI remain the subject of debate. The literature currently suggests that MRI is superior to CT in the diagnosis of isolated ligament injuries [4] and bony bruising [5]. Furthermore, MRI is highly accurate and superior to CT in the diagnosis of isolated trabecular injury [6]. The superiority of MRI over CT in the diagnosis of occult cortical fractures is contentious. Memarsadeghi et al. [7] found CT to be superior in the diagnosis of isolated cortical fractures, and this conclusion is supported by others [8]. Additional considerations are pertinent to paediatric trauma. The radiation dose of an extremity CT scan may be up to 200 times higher than that of a plain radiograph [9] and, in practice, despite publications doubting the significance of a single extremity scan [8], many clinicians prefer to avoid ionising radiation in this population. Furthermore, MRI is advantageous in its ability to diagnose and differentiate between types of Salter–Harris [10] physeal injuries.

The appropriate timing of MRI following paediatric trauma is equally controversial. Current consensus is that MRI should be used to answer or evaluate a specific clinical question or concern rather than as a first-line imaging modality. The primary argument for this is the infrequency of a subsequent enforced changed in management [11], but other authors have added weight to this recommendation by demonstrating the lack of early specificity of MRI, particularly in differentiating undisplaced fractures from bone contusions [5]. Therefore, most authors recommend that MRI should be performed when symptoms persist after a defined period of immobilisation.

The primary objective of this study was to identify the true anatomic pathology sustained in acute paediatric wrist injuries presenting with negative initial radiographs. Secondarily, we sought to compare and contrast our findings with existing adult population data. 75.4 % of our paediatric population were found to have a positive finding on MRI, with a 69.6 % incidence of occult bony injury. The median number of positive findings per patients was 1 (range 0–3), with a 14.3 % incidence of multiple bony injury. In a comparative adult study, Bergh et al. reported an 80 % incidence of MRI abnormality with a median of 2 (range 0–8) positive findings per patient. Pierre-Jerome et al. [12] reported a 62.4 % incidence of occult bone injury with a 68 % incidence of multiple bony injuries. These findings suggest that, whilst the overall incidence of occult bony injury is similar in paediatric and adult populations, children are more likely to sustain localised injuries.

### Fractures

**Fig. 2:**
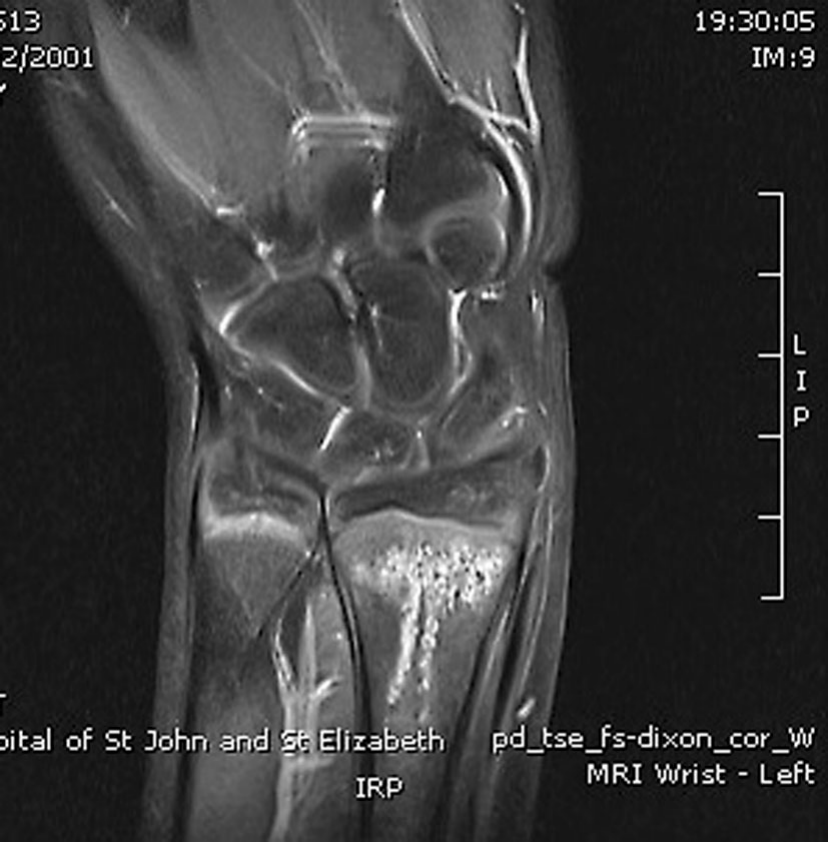
Coronal PD fat sat: intense marrow oedema within the distal radius metaphysis. Small linear area consistent with trabecular fracture

**Fig. 3:**
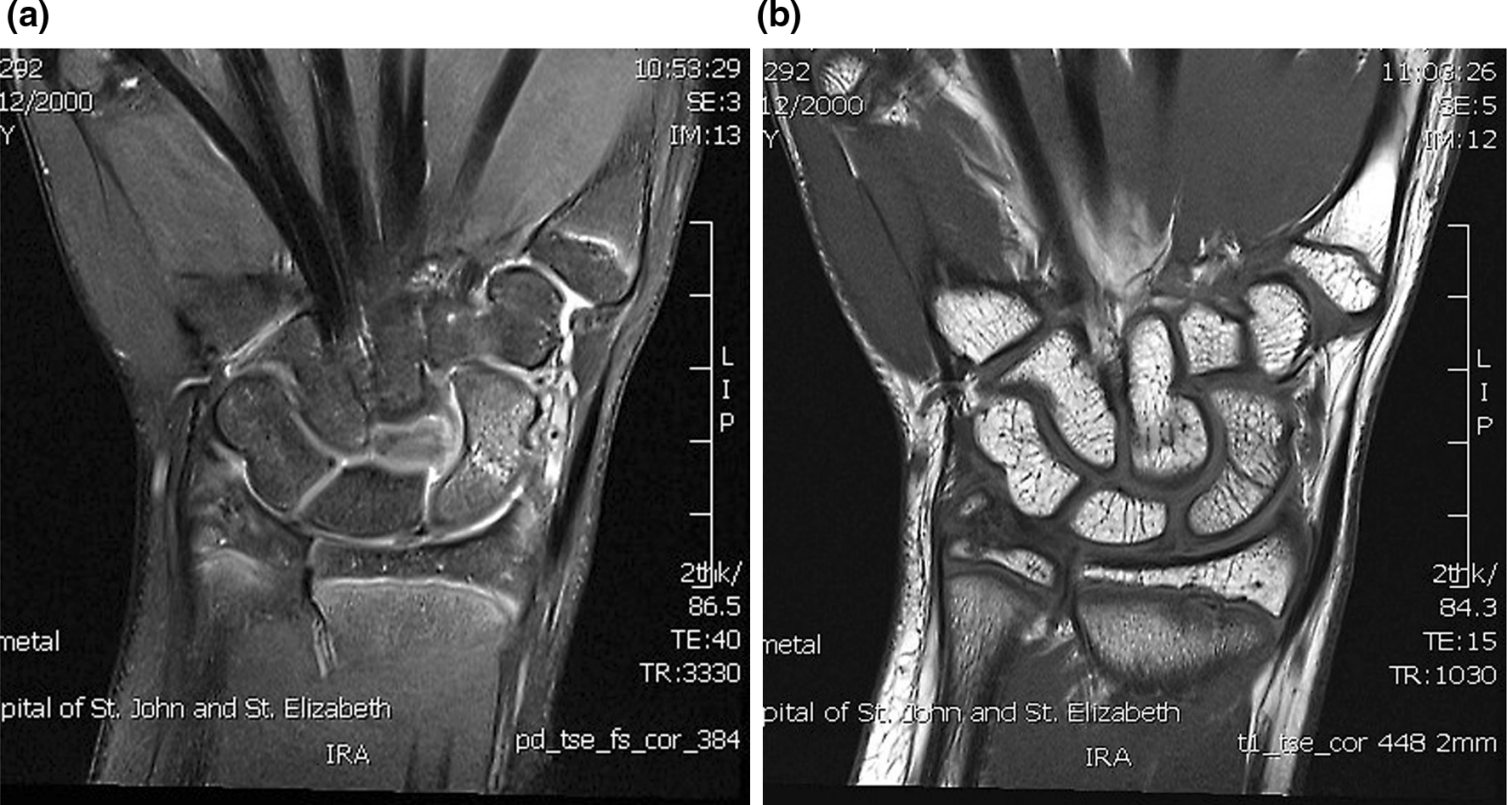
**a** Coronal oblique T1, **b** coronal oblique PD fat sat: undisplaced transverse fracture line extending through the midpole of the left scaphoid. Associated with pronounced bone marrow oedema and periosteal signal change

**Fig. 4:**
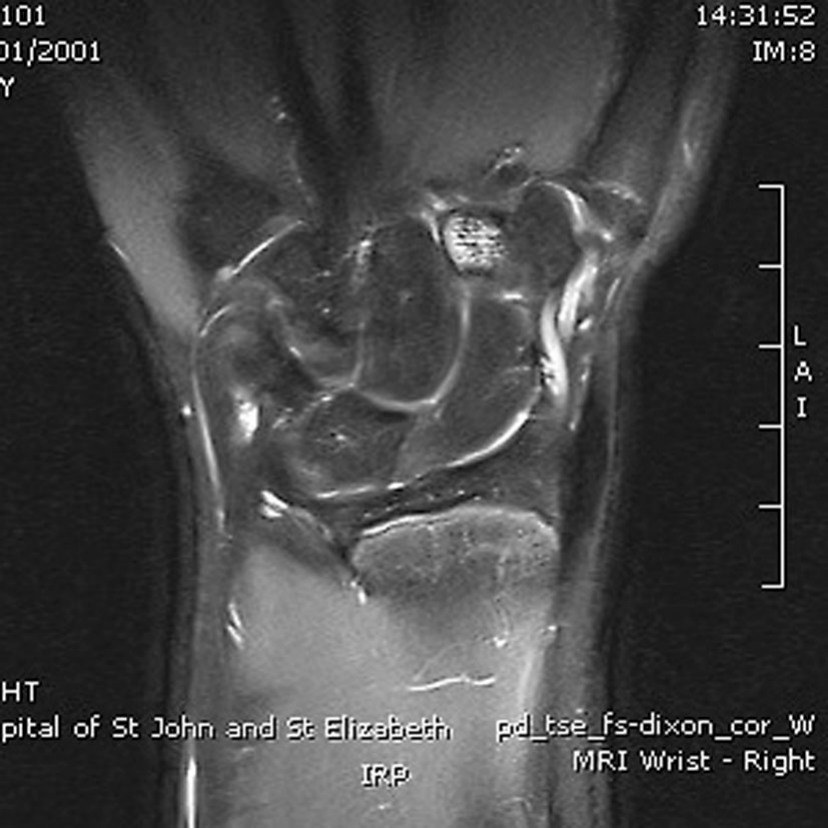
Coronal PD fat sat: bone bruising within right trapezoid bone centered distally and toward palmar surface. This is associated with an undisplaced low signal fracture line

The incidence of paediatric occult fracture was 36.5 % (*n* = 23). Patients with an occult fracture had an average of 1.5 MRI findings. 26.1 % of patients with fractures had an associated bone contusion, 8.7 % had concomitant injuries to the TFCC (1 sprain, 1 tear), and one patient had an injury to the extrinsic ligaments. Further breakdown and comparison is provided in Table 2. In the paediatric population, the distal radius (Fig. 2) was twice as commonly fractured as the scaphoid (Fig. 3a, b), whilst the scaphoid, lunate, and trapezoid (Fig. 4) were the only carpal bones affected. In comparison, Bergh et al. [13] and Pierre-Jerome et al. [12] report an adult occult fracture incidence of 28 and 23.2 %, respectively, with the former study identifying occult fractures of all carpal bones excluding the pisiform and hamate. These results suggest that occult paediatric wrist fractures are twice as likely to occur in the distal radius as they are in the scaphoid, whereas the incidence of occult fracture is equally spread in both bones in the adult population. Furthermore, the number of carpal bones sustaining occult fractures is limited in children in comparison with adults.

**Fig. 5:**
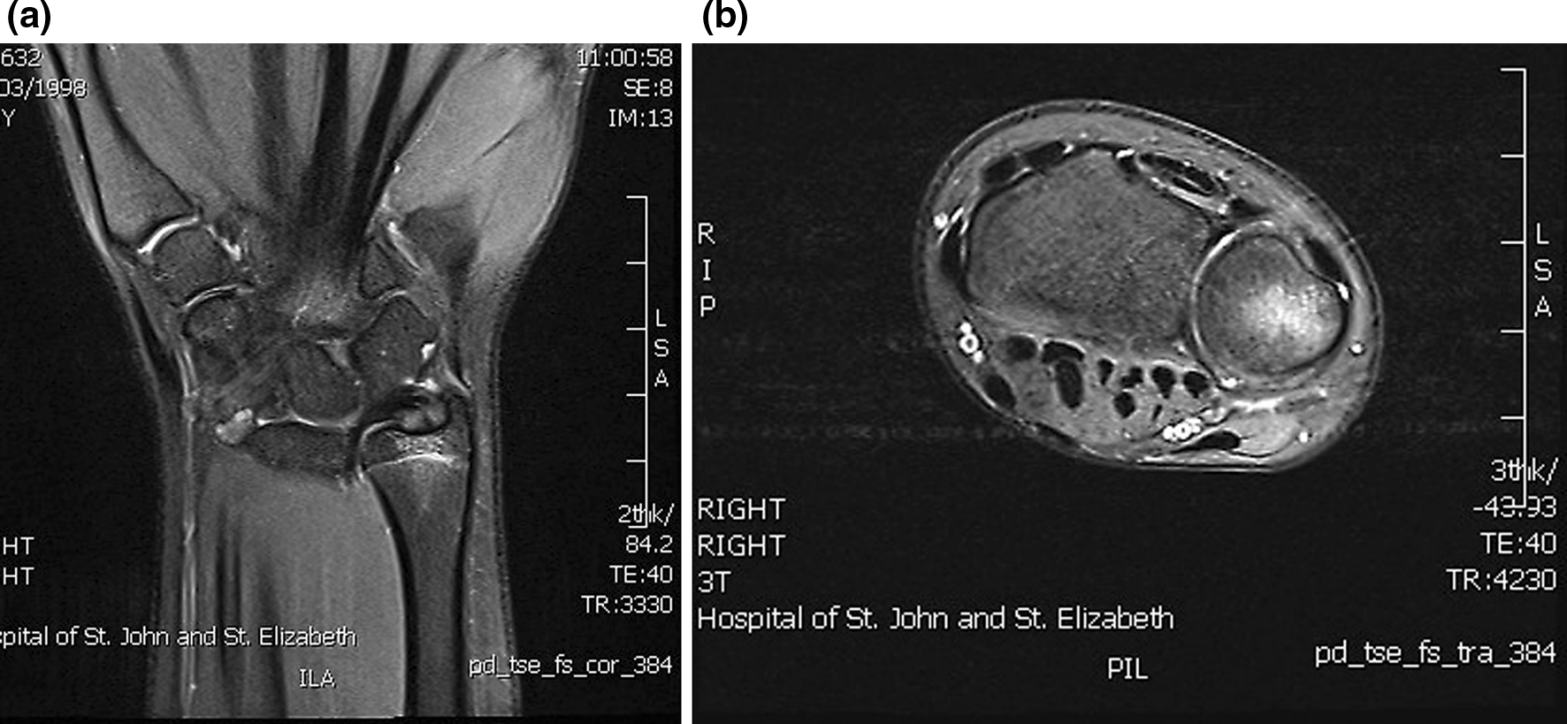
**a** Coronal PD, **b** axial PD fat sat: oedema and widening of distal ulna physis with associated epiphyseal marrow oedema. Findings consistent with Salter–Harris type 1 injury of the distal ulna

### Bone contusion

A bone contusion is defined as focal oedema and haemorrhage of bone secondary to microfracture of the trabeculae, and represents a discrete subtype of bone injury [14]. Their clinical relevance stems from the observation that at 5–6 weeks, where occult cortical fractures are typically asymptomatic due to either a reduction in intraosseous pressure by evacuation of the haematoma from the fractured cortex or an acceleration of the healing process encouraged by the activated periosteum, bone contusions typically remain painful on palpation [5].

The incidence of bone contusion was 35 % (*n* = 22). Bone contusions were observed most frequently in the distal radius (40 %), with further contusions observed in the scaphoid, trapezoid, lunate and ulnar styloid. Multiple contusions were observed in 9 % of cases, and in 28 % of cases a bone contusion was associated with an occult fracture. Sferopoulos et al. [5] have previously classified bone contusions of the distal radius according to their location. Type 1 contusions are localised to the metaphysis and can be thought to represent complete, undisplaced Salter–Harris type 1 fractures. Type 2 injuries involve both the metaphysis and diaphysis, whilst type 3 injuries extend on both sides of the physis and may represent Salter–Harris type 5 injuries. According to this description, 67 % of distal radius contusions found within our study were type 1 contusions, 22 % were type 2, and 11 % were type 3. Further analysis is depicted in Table 2.

In comparison, Bergh et al. [13] and Pierre-Jerome et al. [12] demonstrated an incidence of bone contusion of 22 and 39 %, respectively, with multiple bone contusions observed in up to 60 % of cases. The incidence of distal radius contusion was 14.5 and 23.7 %, respectively, with the contusions seen throughout the rest of the carpus. Overall, paediatric bone contusions appear to affect the distal radius more frequently, whereas there is an equal spread throughout the wrist in adults, where multiple bone contusions are common. Type 1 bone contusions representing complete Salter–Harris type 1 injuries were predominant in our study.

### Soft-tissue injury and ganglia

The incidence of soft-tissue injury (isolated or associated) was 14 %. The range of soft-tissue injuries found on MRI is documented in Table 1. Patients with a soft-tissue injury had a mean of two positive MRI findings. There were no cases of isolated soft-tissue injury, with associated occult bony injuries present in all cases. In comparison, Bergh et al. [13] found an adult incidence of soft-tissue injury of 26.4 %, the most common findings being those of a TFCC injury and partial tendon rupture (see Table 2). In addition, 44.5 % of patients had significant synovitis, and 24.5 % of patients had soft-tissue oedema. Soft-tissue injuries are less common within the paediatric population and, when present, they are in association with occult bony injuries. Eight patients were found to have ganglia on MRI. All of these patients had additional pathology (57.1 % bone contusion, 28.6 % occult fracture, and 14.2 % effusion), and the ganglia were thought to represent incidental findings.

### Diagnosis

The MRI diagnosis differed from the clinical diagnosis in 70.2 % of cases (*n* = 39). The Cohen’s kappa coefficient between initial and final diagnosis was 0.126 (95 % CI 0.040–0.212), suggesting that the strength of agreement is poor, even when the assessment is performed by an experienced hand and wrist consultant. 26.8 % of the population were clinically suspected to have a soft-tissue injury. The MRI incidence was 14.5 %, and all cases were associated with an occult bony injury. The diagnostic challenge was further evident in a comparison of initial management and subsequent MRI findings. One patient (50 %) treated with analgesia alone, 10 patients (29 %) treated with a removable splint and eight patients (40 %) treated with POP had unremarkable MRIs with no positive findings.

### Management

Two patients (clinically suspected soft-tissue injuries) declined a removable splint at initial presentation and were treated with analgesia alone prior to MRI. The remaining study population were treated according to the defined protocol.

At first follow-up post-MRI (median 9 days post initial presentation, range 0–15), patient management was changed in 21 cases (35.1 %). Fifteen patients had their prescribed period of immobilisation reduced or abolished altogether (ten unremarkable MRIs, five bony contusions). Ten of these patients were discharged at their first post-MRI appointment (all unremarkable MRIs). Three patients underwent conversion of the removable splint to POP (two distal pole of scaphoid fractures, one trapezoid fracture). Three patients were prescribed an extended period of immobilisation in POP (two scaphoid waist fractures and one lunate fracture—all protected for a total of 5 weeks). No patients required operative intervention on the basis of the MRI findings. All patients were subsequently discharged with a median time to discharge of 37 days from initial injury (range 7–81), and no complications were recorded.

As a retrospective case series, this study has some intrinsic limitations. Retrospective studies have inevitable selection bias, and in this case the patient group was self-selective in that minor traumas (with minimal findings on physical examination) which were not felt to be serious enough for specialist evaluation may not have been presented to the hand injury unit. Furthermore, the paediatric–adult comparison is based upon heterogeneous data sets.

Nevertheless, this is the first study to define the true anatomic pathology of occult paediatric wrist injuries. It also demonstrates key differences between comparative adult population data. The absence of any isolated soft-tissue injuries suggests that the diagnosis of wrist sprain is inappropriate for use in a paediatric population, and contradicts observations from a previous study investigating paediatric occult ankle injuries where soft-tissue injuries predominated on MRI [15]. Whilst MRI affected a management change in 35.1 % of patients, 30 % of these cases involved a reduced period or complete cessation of immobilisation. We therefore support the consensus that MRI should be reserved to answer a specific clinical concern that remains after a defined period of immobilisation. Based on the findings of this study, our practice of treating acute paediatric wrist injuries with negative radiographs is to explain to patients and parents that these injuries are likely to represent bone bruises or undisplaced stable fractures. Symptoms should typically subside following 3 weeks of rest in a splint. For those that remain significantly symptomatic, and where clinical concern exists, an MRI is offered at this time. The term “wrist sprain” is no longer used in this population. It is our belief that a precise knowledge of the pathology facilitates accurate information delivery whilst reducing parental uncertainty and treatment variation.
